# Stabilizing Spatially-Structured Populations through Adaptive Limiter Control

**DOI:** 10.1371/journal.pone.0105861

**Published:** 2014-08-25

**Authors:** Pratha Sah, Sutirth Dey

**Affiliations:** Population Biology Laboratory, Biology Division, Indian Institute of Science Education and Research-Pune, Pashan, Pune, Maharashtra, India; University of California, Berkeley, United States of America

## Abstract

Stabilizing the dynamics of complex, non-linear systems is a major concern across several scientific disciplines including ecology and conservation biology. Unfortunately, most methods proposed to reduce the fluctuations in chaotic systems are not applicable to real, biological populations. This is because such methods typically require detailed knowledge of system specific parameters and the ability to manipulate them in real time; conditions often not met by most real populations. Moreover, real populations are often noisy and extinction-prone, which can sometimes render such methods ineffective. Here, we investigate a control strategy, which works by perturbing the population size, and is robust to reasonable amounts of noise and extinction probability. This strategy, called the Adaptive Limiter Control (ALC), has been previously shown to increase constancy and persistence of laboratory populations and metapopulations of *Drosophila melanogaster*. Here, we present a detailed numerical investigation of the effects of ALC on the fluctuations and persistence of metapopulations. We show that at high migration rates, application of ALC does not require *a priori* information about the population growth rates. We also show that ALC can stabilize metapopulations even when applied to as low as one-tenth of the total number of subpopulations. Moreover, ALC is effective even when the subpopulations have high extinction rates: conditions under which another control algorithm had previously failed to attain stability. Importantly, ALC not only reduces the fluctuation in metapopulation sizes, but also the global extinction probability. Finally, the method is robust to moderate levels of noise in the dynamics and the carrying capacity of the environment. These results, coupled with our earlier empirical findings, establish ALC to be a strong candidate for stabilizing real biological metapopulations.

## Introduction

Controlling chaotically fluctuating and extinction-prone populations is of major interest to ecologists and conservation biologists and has been an active area of investigation for the last two decades [1]. Although substantial progress has been made in terms of ameliorating chaos in the fields of chemical sciences, physical sciences, electrical engineering, medicine and economics (reviewed in [2], [3]), few strategies have been demonstrated to be successful in stabilizing biological populations. One reason for this is the fact that short and noisy time series typical of most biological populations make it statistically difficult to distinguish noisy limit cycles from chaotic trajectories. Moreover, the parameters of biological populations (e.g. intrinsic growth rate or carrying capacity) are typically estimated *a posteriori* through model-fitting, and are almost never available for perturbation. This precludes the usage of many techniques that demand substantial knowledge of the dynamics of the system (e.g. [4]). To alleviate some of the above-mentioned problems, a number of methods have been proposed that stabilize a system through perturbation of the state-variable, i.e. the population size [5], [6], [7], [8], [9]. For example, it has been theoretically shown that constant immigration can convert chaotic trajectories into limit cycles in spatially homogeneous [8], [10] as well as spatially-structured [5], [7] populations. Similarly, regular perturbation towards a target population size can also reduce the overall temporal fluctuations in the time series [11]. Among the methods that perturb the state-variable, one of the promising strategies in the context of biological populations is the so-called “limiter” family of algorithms. Broadly speaking, the limiter strategy works by not allowing the population size to go above or below some pre-determined threshold, and typically requires some *a priori* information about the dynamics of the system. Although proposed and verified in the context of physical systems like double diode circuits [12], later theoretical investigations have established the generalizability of the concept to other systems (e.g. [13]) including models of population dynamics [14], [15]. However, until recently, there was no empirical support for the efficacy of any of the several limiter control algorithms in the context of biological populations or metapopulations.

Recently, we proposed a novel limiter strategy, called the Adaptive Limiter Control (ALC), to stabilize the dynamics of spatially-unstructured and –structured populations [16]. ALC is a restocking strategy that seeks to maintain populations and metapopulations above a threshold. However, instead of a fixed threshold [14], [15], the magnitude of the perturbation is a function of the difference in the population size in two successive generations. We also empirically demonstrated the effectiveness of ALC in reducing the amplitude of fluctuations in size of replicate laboratory populations and metapopulations of *Drosophila melanogaster*. Interestingly, ALC was able to reduce the extinction probability of the said populations as well. Biologically realistic simulations of ALC using three different non-species-specific models which also incorporated noise, extinction probabilities and lattice effects [17], were able to capture most of the trends of the data. These simulations showed that the obtained experimental results were not due to some idiosyncratic features of the experimental system but are likely to be generalizable. The latter conclusion was bolstered by an analytical investigation suggesting that ALC will always stabilize the dynamics of spatially-unstructured populations represented by unimodal maps [18]. Since the focus of the previous studies were either *in vivo* empirical (i.e. using living organisms like fruit-flies) validation [16] or analysis of spatially-unstructured populations [18], there has been little exploration of ALC in terms of its effects on the dynamics of spatially-structured populations. We aim to address that issue in this paper.

In this study, we further explore the efficacy of ALC *in silico* (i.e. using numerical simulations) in stabilizing the dynamics of metapopulations governed by coupled Ricker maps. We do not seek to establish any analytical results in this paper as such results are typically difficult to attain in the context of spatially-structured, stochastically fluctuating, extinction-prone populations. We demonstrate that compared to an unperturbed system, ALC-controlled metapopulations are more stable over a wide range of intrinsic population growth rate, carrying capacity and extinction probability. The controller is shown to be effective with as low as 1 out of 9 or 10 subpopulations being controlled on both 1-D and 2-D lattices. We find that, barring low migration rates (<20%), ALC either stabilizes metapopulations or fails to have an effect, but never reduces the global stability (measured in terms of metapopulation constancy and persistence) as compared to its uncontrolled counterpart. This implies that one does not require extensive *a priori* knowledge of the parameter values of the metapopulations, which is a definite benefit in terms of the applicability of the method for practical purposes.

## Methods

### Adaptive Limiter Control (ALC)

ALC stabilizes populations by preventing a (sub) population from going below a predefined fraction (*c*) of its size in the previous generation. Since *c* is a fraction and not a fixed number, the method automatically “adapts” to populations inhabiting environments with different carrying capacities or exhibiting an increasing or decreasing trend in size. This method thus includes a mechanism for adjusting the magnitude of the perturbation and hence is related to the “adaptive control” methods in control theory [19]. The population is perturbed only if the current population size falls below the ALC threshold and involves restocking individuals from an external source until the current population size reaches the ALC threshold. The method can thus be represented as:
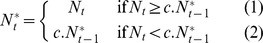
where *N_t_* indicates the population size at a particular generation *t* before the imposition of ALC, N^*^ is the population size post ALC treatment and *c* is the ALC magnitude. Note that N^*^ is also the breeding population size at the end of a generation. Therefore, the population size of the *t+1* ^th^ generation before ALC treatment will be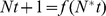. It is known that the time series of *N_t_* and N*_t_ can sometimes exhibit different kinds of dynamics [18], [20]. Here, we restrict ourselves to the dynamics of N^*^_t_ which, being the breeding population size, is more relevant from a biological point of view.

Clearly, setting *c* = 1 and implementing ALC in every constituent population of a metapopulation, has the potential of reducing the dynamics of the system to a fixed point. However, this is practically difficult to achieve due to the extensive intervention effort that would be needed to census and perturb each subpopulation independently. Thus, we focus on the stabilizing efficacy of much lower values of *c* applied to only a subset of subpopulations. Following a previous study [16], the rest of our analysis and discussion focuses on two values of ALC: *c* = 0.25 and *c* = 0.4 which we refer to as Low Adaptive Limiter Control (LALC) and High Adaptive Limiter Control (HALC) respectively. We choose to use these two values again so that the empirical results of the previous study are directly comparable with the corresponding numerical results here.

### Simulations

We used the Ricker equation [21] to examine the asymptotic behaviour of ALC. The Ricker equation is given as (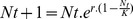) where *N_t_* denotes the population size at time *t*, *r* is the per-capita intrinsic growth rate and *K* is the carrying capacity [22]. First-principles derivations indicate that populations which exhibit scramble competition and random distribution of individuals over space are expected to follow Ricker dynamics [23]. These conditions are likely to be applicable to a wide variety of organisms. Therefore not surprisingly, the model has been shown to be a good descriptor of the dynamics of populations of a large number of taxa including bacteria [24], fungi [25], ciliates [26], crustaceans [27], fruit-flies [28], [29], fishes [21] etc. Thus, insights gained from Ricker-based simulations are likely to be applicable across wide range of organisms, which was the chief reason for our adopting it as a descriptor of the unperturbed dynamics.

Following earlier studies [16], [30], noise was added in every iteration to the *r* parameter in the form of a random number *ε* drawn from a uniform random distribution of range −0.2≤*ε*≤0.2. The final population growth model can thus be represented as: N_t+1_ = N_t_.exp((r+ε).(1-N_t_/K)). In our simulations, unless otherwise mentioned, we consider the value of intrinsic growth rate parameter to be 3.5 which lies within the range of parameter space representing chaotic region in a Ricker map. This value of *r* was chosen primarily due to two reasons. Firstly, the effect of migration rate on the stability of an unperturbed metapopulation is observed primarily when the intrinsic growth rate is high, allowing the dynamics of the neighbouring subpopulations to go out-of-phase [31]. For the Ricker model, this phenomenon is observed in the chaotic zone [30]. Thus, considering chaotic intrinsic growth rates allow us to study the effects of ALC on metapopulations that actually vary in their inherent levels of stability due to different rates of migration [30]. Secondly, we were also interested in observing the interaction of ALC and subpopulation extinction in affecting the stability of the system, and a system with high growth rate is more prone to density-dependent extinctions than one with low growth rates. This is because when growth rates are high, the population is more likely to hit low population sizes, thus increasing the chance of extinction (see next paragraph). We note here that this value of *r* is within the range of estimated growth rates from natural populations [32]. We also explicitly look at the effects of varying the intrinsic growth rate over a large parameter range including stable points, limit cycles of different periodicities and chaos. The initial population size (N_0_) and the carrying capacity (*K*) were arbitrarily fixed at 20 and 30 respectively, unless mentioned otherwise.

The unmodified Ricker model does not takes zero-values and thus theoretical populations (and metapopulations) governed by the Ricker population growth function never go extinct. As subpopulation extinction is known have an impact on the dynamics of metapopulations [30], [33], we explicitly introduced stochastic extinction in our models by implementing an extinction probability of 0.5 below a threshold population size of 4 [16], [30]. In other words, P(N_t_ = 0 | N_t_′< = 4) = 0.5 (unless explicitly mentioned otherwise). Here, *N*_t_′ denotes the post-migration population size of the *t^th^* generation while *N_t_* denotes the population size after the extinction step but before the implementation of ALC. On the event of metapopulation extinction, the metapopulation was reset with a population size of 8 per subpopulation. Note that for *c*>0, implementation of ALC ensures that the metapopulation size is more than zero at each generation. Thus, only the unperturbed (*c* = 0) metapopulation were reset during extinction events.

For simulations in this study, a metapopulation is described as two or more subpopulations connected to each other via symmetric rate of migration, i.e. the immigration and emigration rates for all patches were equal. For metapopulations consisting of more than two subpopulations, the subpopulations were considered to occupy spaces on the periphery of an imaginary circle so that dispersal occurred between the two nearest neighbours of a subpopulation. In other words, the system is a one-dimensional lattice with periodic boundary conditions (a finite ring). In nature, such metapopulations can be found along the edge of a lake or a park. In perturbed metapopulations, ALC was imposed after migration so that immigration due to ALC for a given generation would have an impact on the population size of the neighbour only through migration in the subsequent generation. Except the case where we study the effects of perturbing different fractions of subpopulations, ALC was always applied to only one subpopulation in the metapopulation.

The complete sequence of steps in the simulations was as follows:

N_t-1_* → *f*(N_t-1_*) → [migration] → *N_t_′*→[extinction] → *N_t_* → [ALC] → N_t_*

where N_t-1_* is the subpopulation size after the application of ALC in generation *t-1*; *f*(N_t-1_*) denotes the population size post-reproduction (i.e. application of the Ricker function) on which we impose the migration operation to get population size *N_t_′*. Extinction, if applicable, is imposed at this stage which yields the pre-ALC value of *N_t_*. This value of *N_t_* is then compared with N_t-1_* to determine whether ALC is to be applied or not (as per equation 1, 2), leading to the final breeding population size of N_t_*. When *c* = 0, an extinct population is reset to a value of 8. The metapopulation size of a given generation *t* is computed as the sum of the N_t_*s for all the subpopulations and indices to measure global stability are calculated on this metapopulation size.

All simulations were run using MATLAB R2010a (Mathworks Inc.) and each point in the figures represents an average of 100 simulation runs. The error bars represent standard error of the mean. The first 400 iterations of each run were rejected, and all indices of stability (see next section) were computed over the next 100 iterations.

### Stability properties and synchrony

The definition of stability happens to be controversial and a 1997 review enumerates no less than 163 definitions pertaining to 70 different stability concepts in the ecological literature alone [34]. Following the nomenclature of [34], we have considered the efficacy of ALC w.r.t two kinds of stability properties of the metapopulation time-series, namely constancy and persistence. Constancy is defined as the property of a system to stay essentially unchanged [34]. We quantified constancy using a widely used [35], [36] measure called the Fluctuation Index (FI):
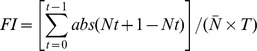
where 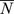
and *T* stand for the average population size and total number of generations respectively. FI is a dimensionless quantity which measures one-step fluctuation in population or metapopulation size across generations, scaled by the mean population size [30]. High FI implies reduced constancy and vice versa.

Persistence was quantified as metapopulation extinction frequency, i.e. the average number of generations a metapopulation records a zero population size (before the application of ALC) as a proportion of the total number of generations. Thus, high extinction frequency of the system indicated the system to be less persistent. We calculated synchrony as the cross-correlation coefficient at lag zero of the first-differenced log-transformed values of the two subpopulation sizes [37].

## Results and Discussion

### Effects of migration rate on constancy

**Figure 1:**
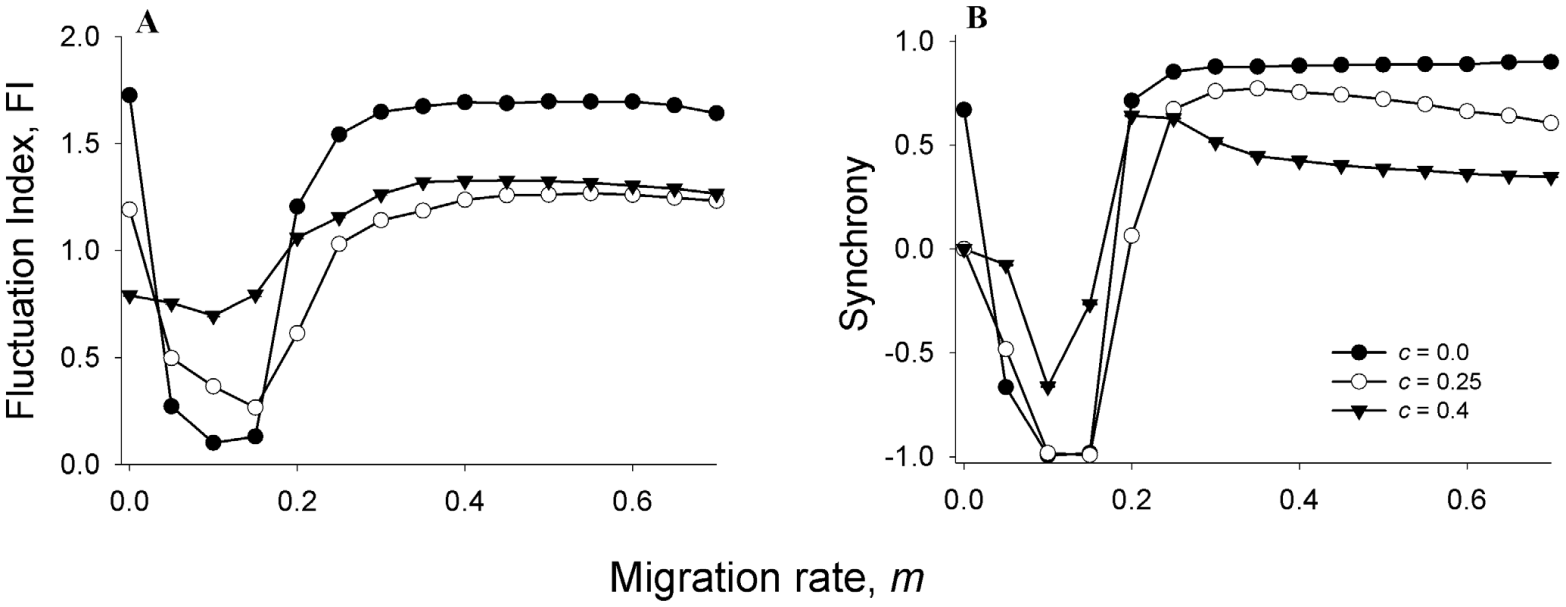
Effects of ALC on metapopulation FI and synchrony at different rates of migration. (**A**). Both LALC (*c* = 0.25) and HALC (*c* = 0.4) increases metapopulation FI at low migration rates, but reduces the same at high migration rates. This contrasting effect can be explained by (**B**) which shows that ALC reduces both positive and negative synchrony, which in turn is expected to have opposite effects on metapopulation constancy. *N_0_* = 20 and *K* = 30 for all figures including this one. Intrinsic growth rate, *r* = 3.5 (for this and all subsequent figures except Figure 2). Each point in every figure is a mean of 100 independent runs. Error bars denote ±SEM and are too small to be visible.

The rate of migration between subpopulations is known to influence the dynamics of metapopulations [30], [31], [38] and thereby can have a major impact on the efficacy of a control technique [16]. We therefore tested the effect of ALC on the constancy of two-patch metapopulations at different rates of symmetric migration. We find that compared to the unperturbed case (*c* = 0), both LALC (*c* = 0.25) and HALC (*c* = 0.4) increase the Fluctuation Index (FI) of the metapopulation at low (<20%) rates of migration (Fig. 1A). However, when migration rates are higher, the situation reverses and both levels of ALC seem to reduce the metapopulation fluctuation index. The explanation for this phenomenon lies in the way ALC affects the synchrony between the constituent subpopulations (Fig 1B). In an unperturbed system (*c* = 0), low rates of migration (<20%) reduces metapopulation FI by inducing out-of-phase fluctuations (i.e. negative synchrony) between neighboring sub-populations [30]. This happens because negative synchrony ensures that crashes in some subpopulations are accompanied by booms in others. This in turn reduces the temporal variation in the metapopulation size [30], [31], [38], which by definition, is the sum of all subpopulation sizes. Conversely, in-phase fluctuations between subpopulations at high migration rates reduce constancy (i.e. increase FI) at the metapopulation level, by bringing the subpopulations in phase with each other. It has been earlier shown that ALC reduces both positive and negative synchrony between subpopulations [16], which has contrasting effects on constancy stability. While the reduction of positive synchrony (Fig 1B) reduces metapopulation FI, the lowering of negative synchrony at low migration rate increases metapopulation FI, leading to the observed opposite effects of ALC at the two migration rates (Fig 1A).

It is clear from Fig 1A that an unperturbed system has high FI at higher rates of migration. Since ALC is a perturbation strategy to stabilize an unstable population [16], [18], we focus on a particular rate of migration ( = 30%) for the rest of our investigation. The dynamics at this particular rate of migration has been extensively investigated in previous studies on metapopulations of *Drosophila melanogaster* [16], [30], [33], is known to induce high values of metapopulation FI [30], [33] and is within the range of migration rates experienced by natural populations [39].

### Effects of *r* on constancy and persistence

**Figure 2:**
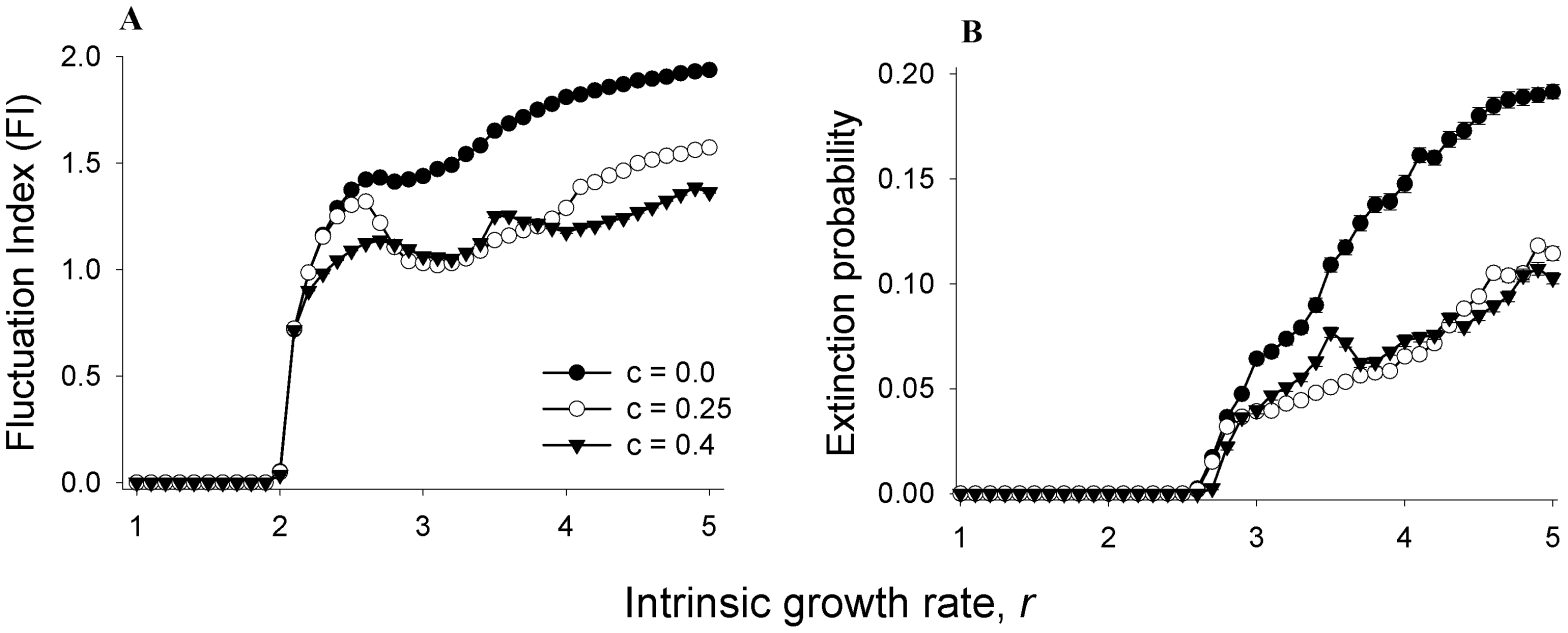
Effects of ALC on metapopulation stability at different intrinsic growth rate (*r*) values. ALC enhances (**A**) constancy and (**B**) persistence over a wide parameter range, and has no effects in other zones. See main text for a possible explanation. Migration rate (*m*) = 0.3 in this and all subsequent figures. Error bars denote ±SEM and are too small to be visible.

Estimating the precise values of parameters like growth rate or carrying capacity is typically difficult for any real population, and often a control strategy will need to be applied without much prior information about the dynamics of a system. Under such conditions, a control method that is known to stabilize metapopulations only under a narrow parameter zone is expected to be of limited use. As part of our investigations on its applicability, we tested the efficacy of ALC in terms of both constancy and persistence at various magnitudes of *r* (+ noise, ε drawn from a uniform random distribution of range −0.2≤ε≤0.2) for the subpopulations (Fig 2). HALC reduces metapopulation FI for all values of *r*>2.2 whereas LALC is effective at a slightly higher range of *r* (>2.7) (Fig. 2A). ALC had no discernible effect on the dynamics at *r*<2.2. These observations can be explained by an interaction of the nature of ALC and the Ricker dynamics. The Ricker model is known to follow a period-doubling route to chaos with the amplitude of oscillation of the population size becoming larger with increasing *r* [40]. ALC perturbations happen only when the population size in a given generation is less than a fraction *c* of its previous generation. In the Ricker model, *r*<2.0 always leads to a stable point cycle, as a result of which, the ALC perturbation is never applied, and the dynamics of the unperturbed population is indistinguishable from the ALC-controlled ones. When *r* lies between 2.0 and 2.2, the system undergoes small amplitude two-point limit cycles in which the population crashes are not sufficiently large to lead to the application of ALC. Thus, again, there is no difference between the control and the perturbed populations. It is only when the amplitude of the limit cycles become sufficiently large (*r*>2.2 in this case) that ALC perturbations are actually applied and there is an effect of the perturbation on FI. Not surprisingly, this reduction of FI is visible for lower values of *r* for HALC (*r*>2.2) compared to LALC (*r*>2.7). This is because the minimum amplitude of the crash needed for ALC to be applied is 60% and 75% of the previous population size for HALC and LALC respectively (as HALC and LALC is imposed only in cases where the current population size is less than 40% and 25% of previous generation, respectively). This implies that, compared to LALC, HALC perturbations begin at lower values of *r* and therefore the stabilizing effect also manifests earlier (Fig 2A). Clearly, these observations should be applicable for all models that follow a period-doubling route to chaos like the logistic [41] or the Hassell [42], although the exact values of the growth rate parameter where ALC becomes effective as a stabilizing factor, would differ. Since the nature of the dynamics (i.e. stable point, limit cycle or chaos) of a Ricker model is independent of the carrying capacity (*K*), our reasoning suggests that the effect of ALC would also remain unaffected by the values of *K*, which was indeed found to be the case (Fig. S1). The interaction between the intrinsic growth rate and the ALC magnitude in determining the metapopulation FI is investigated more thoroughly in Fig S2.

We also investigated the persistence of ALC-perturbed metapopulations, as preventing extinction can be a more pressing objective under certain scenarios. Although ALC was in general effective in reducing the global extinction probability, we did not find a good correspondence between FI (Fig 2A) and the corresponding extinction probability (Fig 2B) for different values of *r.* This was consistent with previous empirical findings that indicate constancy and persistence are often uncorrelated [43], [44]. The reason behind enhanced persistence of ALC-controlled populations lies in the ability of ALC to reduce positive synchrony [16], which in turn reduces the extinction probability of connected subpopulations [45], [46]. This is because a high positive synchrony between subpopulations causes them to reach lower levels simultaneously, reducing the chances of each subpopulation to receive immigrants from their neighbors and thereby increasing the chances of local (and global) extinction. A reduction in positive synchrony by ALC desynchronizes the fluctuation of neighboring populations, which ensures that whenever a population reaches a low size, it is often “rescued” from extinction by immigrants from neighbors with higher population size. This phenomenon of reduced synchrony due to ALC going hand-in-hand with enhanced persistence, has already been observed in the dynamics of *Drosophila* metapopulations [16]. Here we provide a numerical corroboration of the earlier result using biologically realistic simulations.

It should be noted here that ALC also reduces the extinction probability of single populations [16], which can conceivably reduce the metapopulation extinction probability. Although global extinction probability can also be potentially affected by a change (here reduction) in local extinction probability, it is not intuitively obvious how. This is because it is known that local extinctions can affect synchrony (and hence metapopulation persistence) in rather complex ways [47], [48], particularly when the growth rates are high. We are not aware of any study that has partitioned the relative contributions of synchrony and local extinction on metapopulation persistence. While it would be of considerable interest to ecologists, figuring this out is clearly beyond the scope of the current study.

### Local extinction probability and constancy

**Figure 3:**
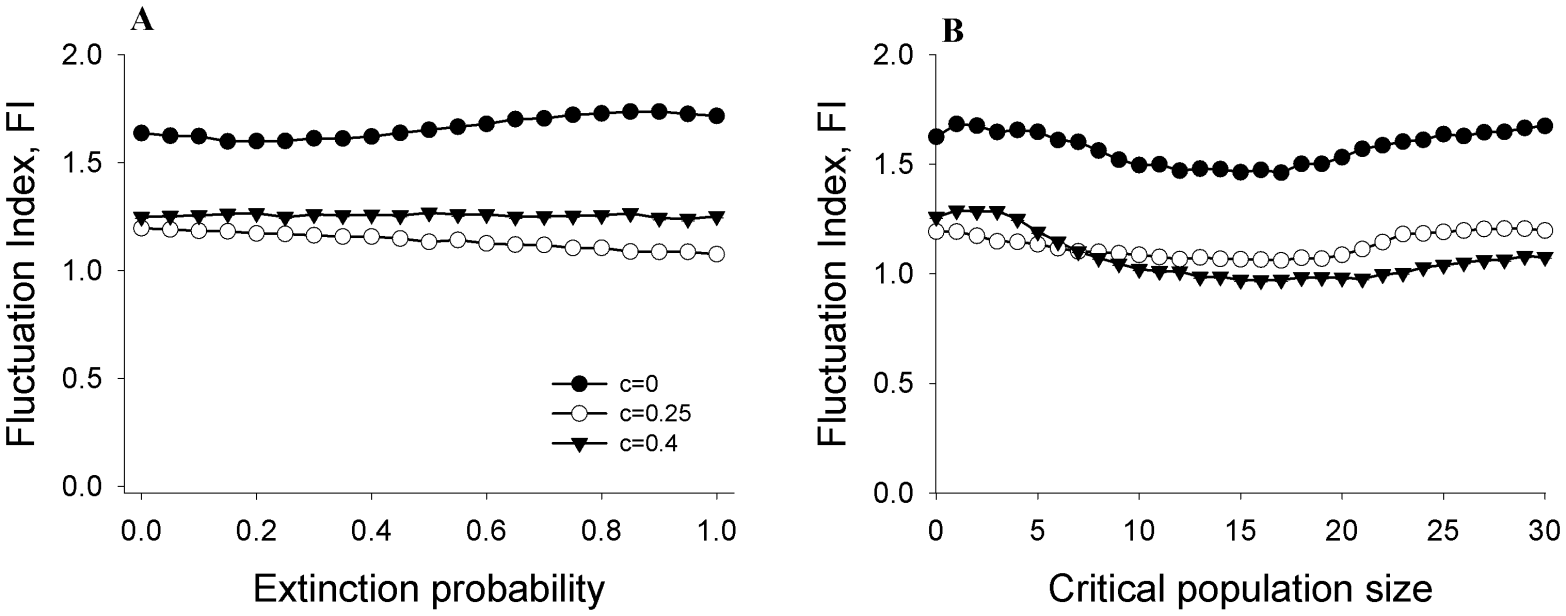
Effects of ALC on metapopulation constancy under different rates of subpopulation extinction. (**A**). With increasing extinction probability when the population size goes below 4. (**B**) With increasing critical population sizes below which, there was a 50% extinction probability that the population would go extinct. In both cases, increasing the rate of extinction did not reduce the efficacy of ALC in inducing greater constancy. See main text for a possible explanation. Error bars denote ±SEM and are too small to be visible.

The subpopulations of a metapopulation often go extinct [49], [50], which in turn can play a role in determining the dynamics of the system [47], [51]. Such local extinctions can also modulate the effects of a control strategy. For example, local extinctions were implicated when constant immigration [7], [52] was found ineffective in stabilizing laboratory metapopulations of *Drosophila melanogaster* [33]. Although ALC has been empirically demonstrated to be effective in stabilizing the dynamics of extinction-prone populations [16], a detailed investigation of the effects of local extinction on metapopulation stability is lacking. In the simulations of the previous section, we had assumed a particular probability of extinction ( = 0.5) when a subpopulation touched or fell below the critical population size threshold of 4. We therefore investigated the performance of ALC for different values of the extinction probability of the subpopulations each time they reached or went below a threshold of 4. We found that increasing the probability of subpopulation extinction did not reduce the ability of ALC to induce metapopulation stability (Fig. 3A). This observation also held true on varying the threshold of critical population sizes keeping a constant extinction probability of 0.5 (Fig 3B). These observations indicate that, unlike constant immigration [33], ALC is capable of reducing the FI in the presence of a range of subpopulation extinction probabilities. The robustness of ALC towards subpopulation extinction probability can be explained by the very way in which ALC is designed: in the time series whenever there are population crashes (including extinctions), ALC brings the population size back to a higher number. Thus, extremely low values of the breeding-population size are never permitted irrespective of the extinction probability or the critical threshold. This reduces the magnitude of both the population crashes as well as the subsequent spikes, which in turn contributes to the reduction in Fluctuation Index.

### Larger metapopulations and larger fraction of controlled patches

**Figure 4:**
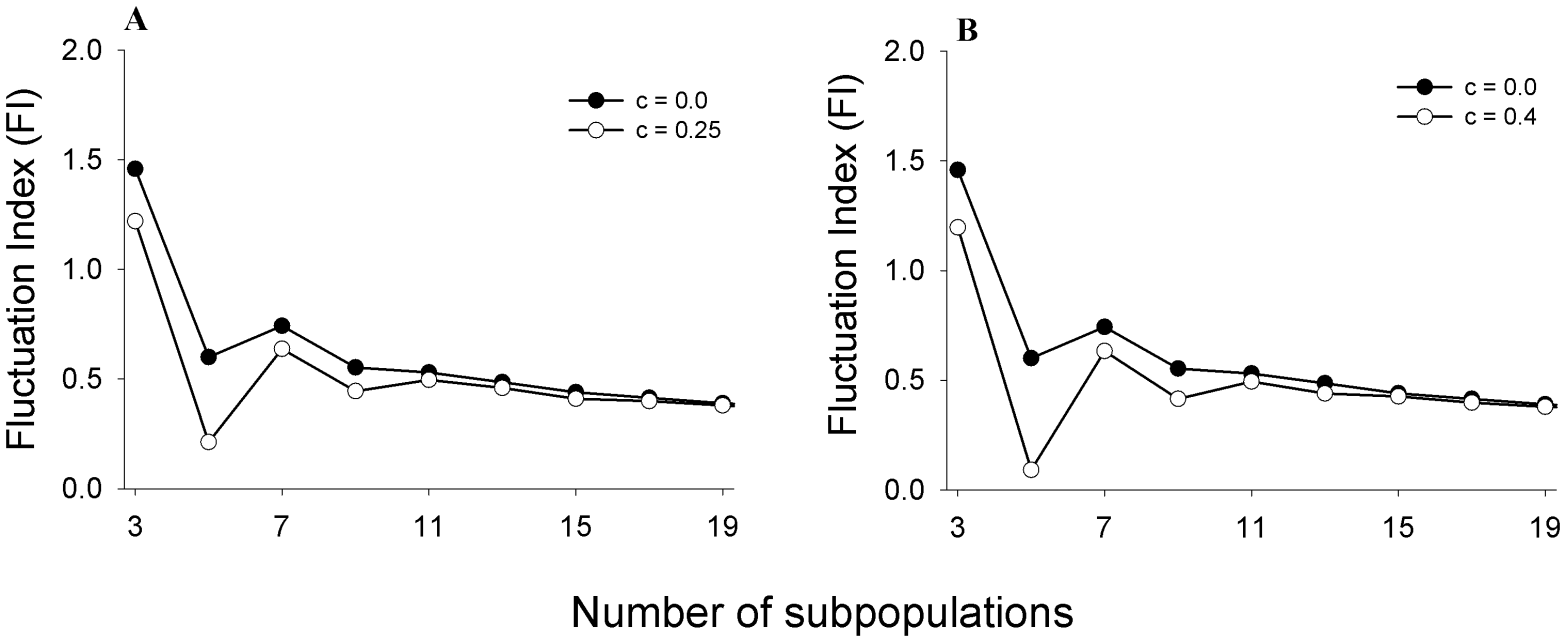
Effects of ALC on constancy in metapopulations with different number of subpopulations. (**A**) LALC (i.e. c = 0.25), and (**B**) HALC (i.e. c = 0.4). In both figures, only one subpopulation is perturbed for increasing number of subpopulations. Perturbing only 1 patch by ALC can reduce FI of metapopulations with up to 10 subpopulations. Error bars denote ±SEM and are too small to be visible.

**Figure 5:**
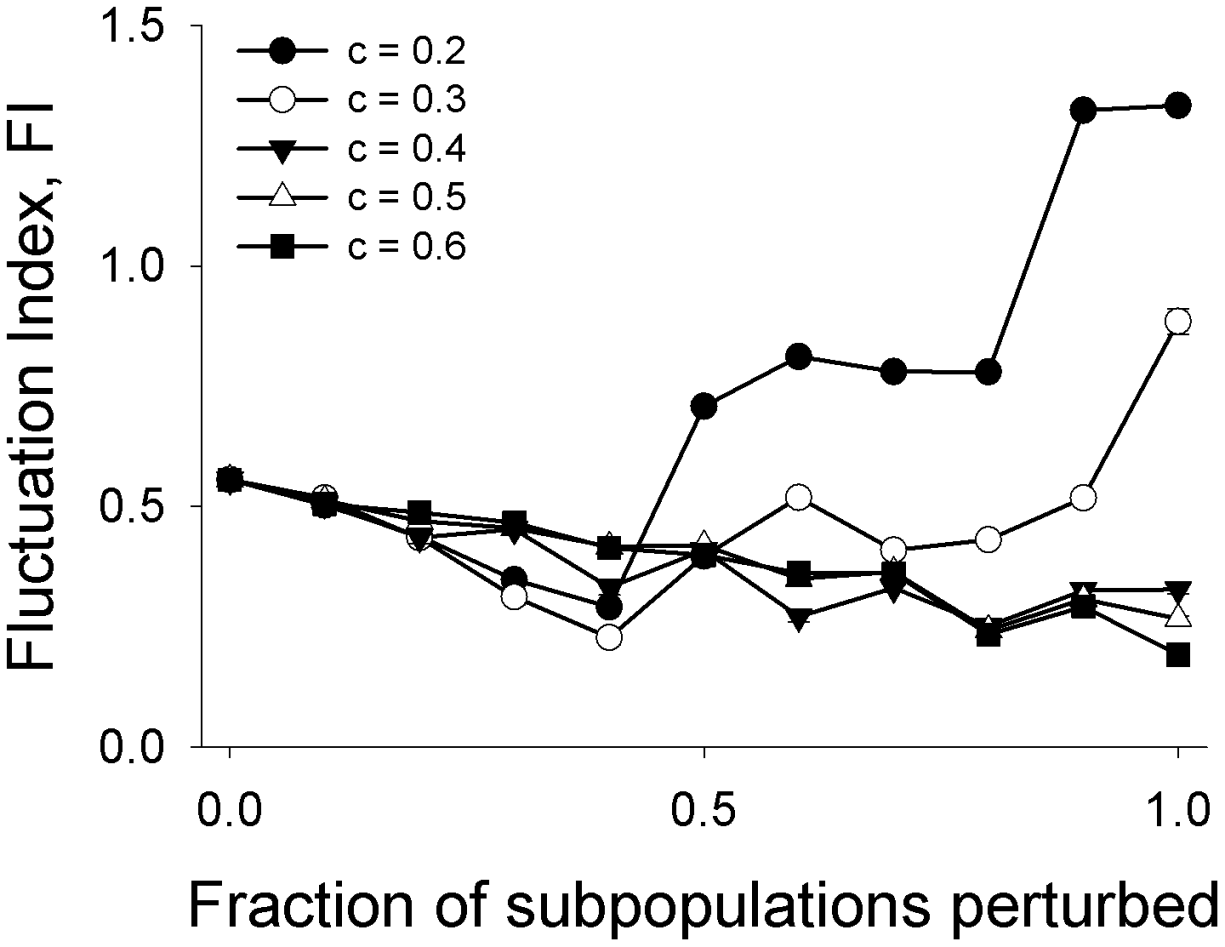
Effects of increasing the fraction of ALC controlled subpopulation on metapopulation constancy. In this figure, each metapopulation consists of 10 subpopulations. For low values of *c*, increasing the fraction of perturbed subpopulations can have a negative effect on constancy. Error bars denote ±SEM and are too small to be visible.

So far we have investigated the adaptive limiter control mechanism on a simple metapopulation consisting of only two subpopulations. However, it is known that metapopulation dynamics can be considerably influenced by the number of constituent subpopulations [53]. Moreover, in our study, we have perturbed 50% of the subpopulations (i.e. one out of two), a fraction that might be difficult to achieve in larger metapopulations in practice. We therefore tested the ability of ALC to stabilize larger metapopulations with only one of the constituent subpopulations being subjected to ALC control. Given that only one patch in the whole metapopulation is being controlled, intuitively, the efficacy of ALC should go down sharply as the total number of subpopulations increases. Surprisingly, ALC perturbed metapopulations with up to 10 subpopulations were still more stable than their unperturbed counterparts (Fig 4A and 4B), with the effect being most pronounced for number of patches ≤5. The FI of an ALC-controlled metapopulation becomes equivalent to an unperturbed metapopulation when there are more than ten subpopulations. This is not surprising since the fraction of perturbed subpopulations (<0.1) is too little to affect the dynamics at a global scale. Perturbing large number of subpopulations with LALC should be avoided as the global FI increases with increasing number of patches for *c*∼0.3 (Fig 5). This observation is consistent with an earlier finding that under constant immigration, increasing the fraction of perturbed patches leads to an increase in metapopulation FI [33]. This implies that the efficacy of a control algorithm can be context-specific and “more control” does not always translate into “better control”. This phenomenon was also observed when ALC was applied to a 2-D 3×3 lattice with periodic boundary conditions (Fig S3A). ALC was able to reduce the metapopulation FI even when only one out of the nine subpopulations was perturbed. Even greater reductions in global FI were obtained on increasing the fraction of perturbed subpopulations to two out of nine and three out of nine. However, when four subpopulations were perturbed, the reduction in metapopulation FI was comparatively less, compared to the 3/9 case. It should be noted here that for a 2-D lattice, the efficacy of a control algorithm can depend on the precise distribution of the perturbed subpopulations in the lattice [7], [54]. For each of the four fractions reported in Fig S3A (i.e. 1 or 2 or 3 or 4 patches out of nine), we simulated multiple patch distributions. Although there were some quantitative differences in the patterns of reduction of metapopulation FI for different distributions (e.g. Fig S3B), the broad trends remained the same: ALC was in general able to enhance global constancy even on a 2-D lattice.

### Closing Remarks

The dynamics of spatially-connected populations are crucially dependent on the life-history of the organisms [55], which in turn can potentially affect the efficacy of a control algorithm. In this paper, we explore this aspect and show how the magnitude of the control parameter (*c*) interacts with the intrinsic growth rate, to determine the constancy and persistence stability of the metapopulation. At a practical level, our main message is that as long as migration rates are high, ALC can either enhance the metapopulation constancy and persistence or have no effect, but can never reduce the stability of the metapopulation to a level less than that of the corresponding unperturbed case with unstable dynamics. This indicates that precise *a priori* knowledge of the growth rate/localized extinction rates and carrying capacity of the constituent subpopulations are not really needed for the application of ALC. It has recently been shown that given certain conditions, ALC can reduce the fluctuations of any 1-D map that has a single-humped first return map and a unique carrying capacity [18]. Since several models of population dynamics, including the widely used Logistic [41] and Hassel [42] models, would satisfy these conditions, our results are generalizable to these systems (Fig S4). However, many organisms that appear on conservation lists (like reptiles, birds, mammals) may not satisfy these conditions or their dynamics may not be well-represented by such 1-D models. Therefore, ALC should not be applied to such species, until and unless it is shown to have a stabilizing effect in the context of the appropriate dynamics. This study did not take into account certain aspects of the dynamics that can potentially affect the efficacy of a control method. For example, although ALC was found to be robust to varying degrees of noise to intrinsic growth rate (Fig S5), natural populations are typically also exposed to stochasticity in environments, interactions with other species etc. Similarly, the scheme of migration [56] and the form of density dependence [57] are known to affect metapopulation dynamics, two factors which were not investigated in this study. Therefore, any attempts to use ALC to control real populations should be based on relevant information about the biology of the organism. Finally, although we show that ALC can increase metapopulation stability under several biologically relevant scenarios, we do not compare its efficacy under those scenarios against other control methods proposed in the literature. Incorporation of parameter noise, local extinctions and spatial structuring typically makes such problems analytically intractable. On the other hand, numerical comparisons are valid only under common conditions, i.e. when the dynamics of the same model, under similar parameter values are compared for similar aspects of stability. Although such a comparison has recently been attempted in the context of spatially-unstructured populations [58], to the best of our knowledge, there exists no such comparison in the context of metapopulations, and might be a fruitful area for future investigations.

## Supporting Information

Figure S1 **Effects of ALC on metapopulation constancy at different magnitudes of carrying capacity.** There was no effect of carrying capacity on the stabilizing efficiency of ALC. Error bars denote ±SEM and are too small to be visible.
(TIF)

Figure S2 **FI of metapopulation with 2 subpopulation as a function of intrinsic growth rate (** ***r*** **) and ALC magnitude (** ***c*** **).**
(TIF)

Figure S3 **FI of 9-patch 2-D metapopulations for different ALC magnitude (** ***c*** **).** In these simulations, the 9 subpopulations were arranged on a 3×3 2-D lattice with periodic boundary conditions for migration. The inset gives the identity of the individual subpopulations. Each subpopulation exchanged migrants with four neighbours (above, below, right and left). Thus, subpopulation #5 exchanged migrants with subpopulation # 2,8,6,4 and so on. With periodic boundary conditions, the subpopulations thus inhabit the surface of a 3-D torus. The migration rate, initial population size, *r*, and K were 0.3, 20, 3.5 and 30 respectively. All other conditions were similar to the 1-D migrations. Each point is a mean of 100 independent simulations and error bars denote the corresponding SEM. a) Metapopulation stability for different number of perturbed subpopulations (identity of subpopulations in bracket) at different values of *c*. ALC was able to stabilize metapopulations in general, even when applied in only 1/9 subpopulations. However, perturbing too many subpopulations leads to a lesser reduction in global FI. b) Metapopulation stability when three subpopulations are perturbed but in different arrangements. Note that although there is an overall decrease in metapopulation FI, the trends and magnitude of decrease are different.
(TIF)

Figure S4
**Effect of ALC on two-patch Logistic and Hassell metapopulations.** (a) Effects of ALC on metapopulation FI using (a) coupled logistic map (x* = r.x(1-x)*, *r* = 0.4, *x_0_* = 0.1) and (b) coupled Hassell map (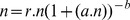; *a* = 0.6, *b* = 10, *no* = 0.4, *r* = 40) at different migration rates. Both LALC (*c* = 0.25) and HALC (*c* = 0.4) reduces metapopulation FI at all migration rate values tested. Thus qualitatively, the effects of ALC on coupled logistic and Hassell map are comparable to those from Ricker (*cf* Fig 1A of the main paper).(TIF)

Figure S5 **Effects of noise in growth rate on constancy of two-patch metapopulations.** In this figure, each metapopulation consists of 2 subpopulations. Noise term (*ε*) represents the magnitude of noise associated with intrinsic growth rate (*r*) in the simulations. Both LALC (*c* = 0.25) and HALC (*c* = 0.4) are robust to varying degrees of noises. Migration rate, *m* = 0.3. Each point is a mean of 100 independent simulations. Error bars denote ±SEM and are too small to be visible.
(TIF)

File S1 **Matlab Code for the simulations in this paper.**
(M) Click here for additional data file.
