# Supplementary figures and images for: Stabilizing Spatially-Structured Populations through Adaptive Limiter Control
Source: PLoS One. 2014 Aug 25;9(8):e105861. doi: 10.1371/journal.pone.0105861 (PMC4143321; doi:10.1371/journal.pone.0105861)

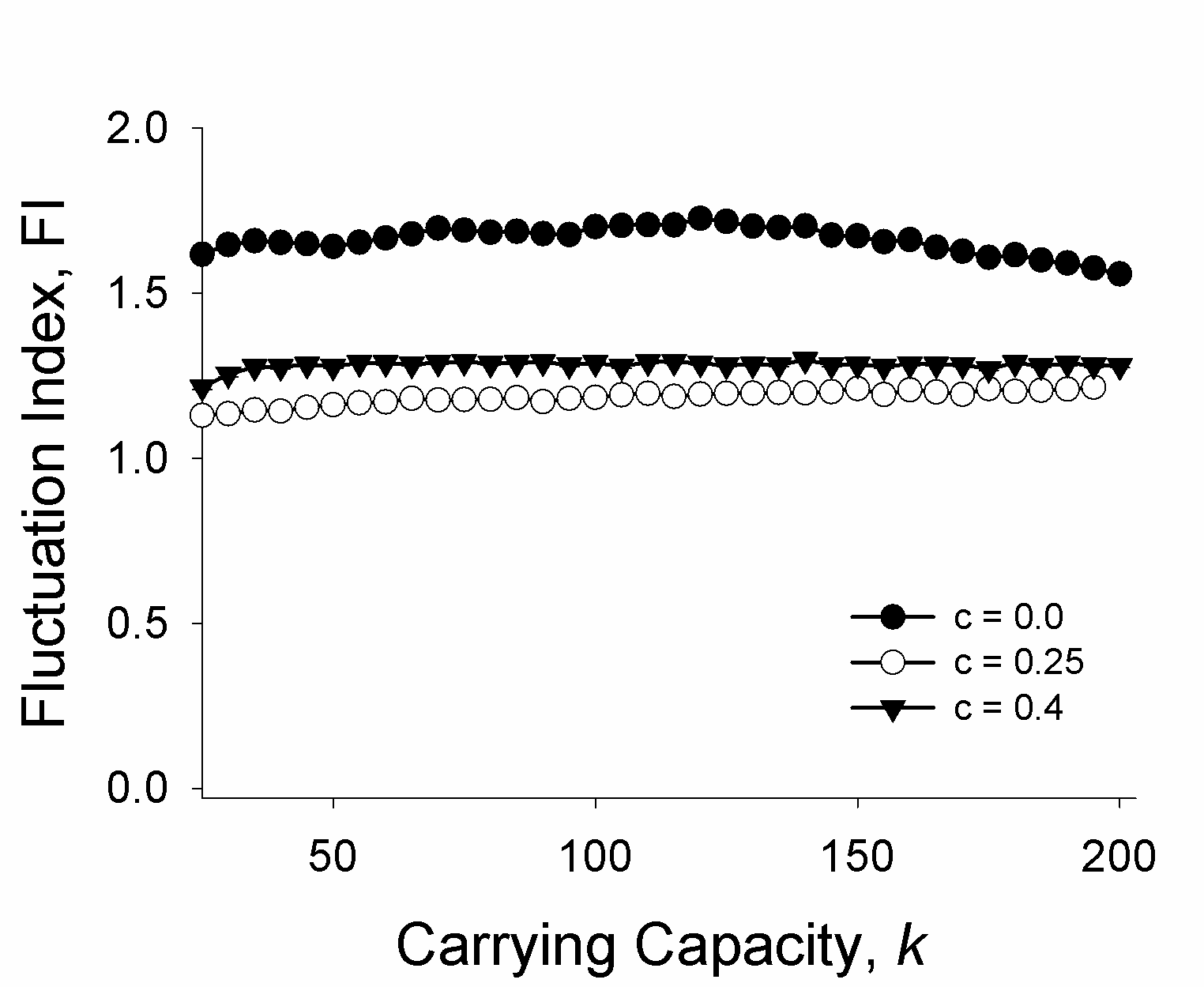

Supplement: Figure S1 — Effects of ALC on metapopulation constancy at different magnitudes of carrying capacity. There was no effect of carrying capacity on the stabilizing efficiency of ALC. Error bars denote ±SEM and are too small to be visible. (TIF) [file pone.0105861.s001.tif]

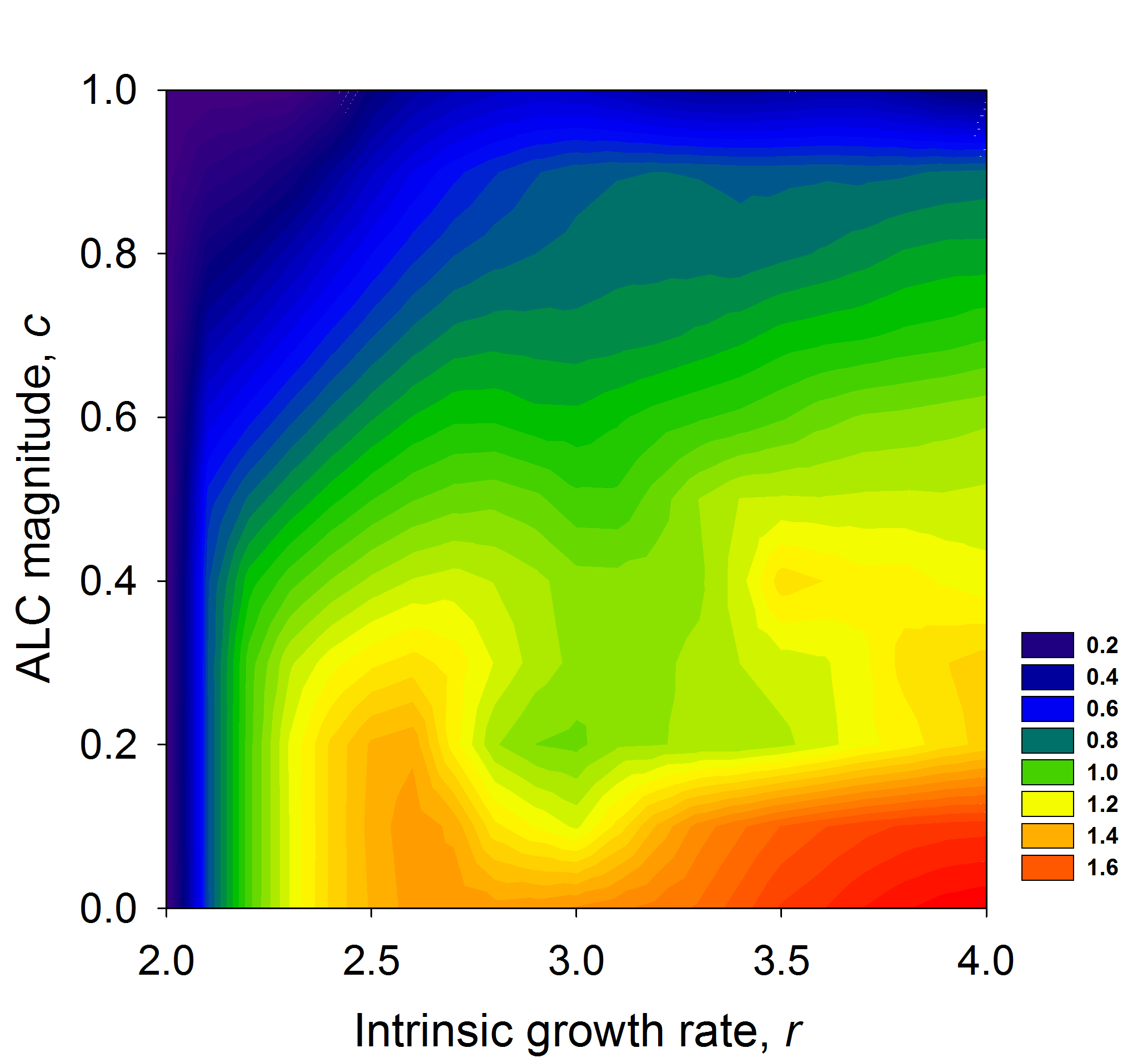

Supplement: Figure S2 — FI of metapopulation with 2 subpopulation as a function of intrinsic growth rate ( r ) and ALC magnitude ( c ). (TIF) [file pone.0105861.s002.tif]

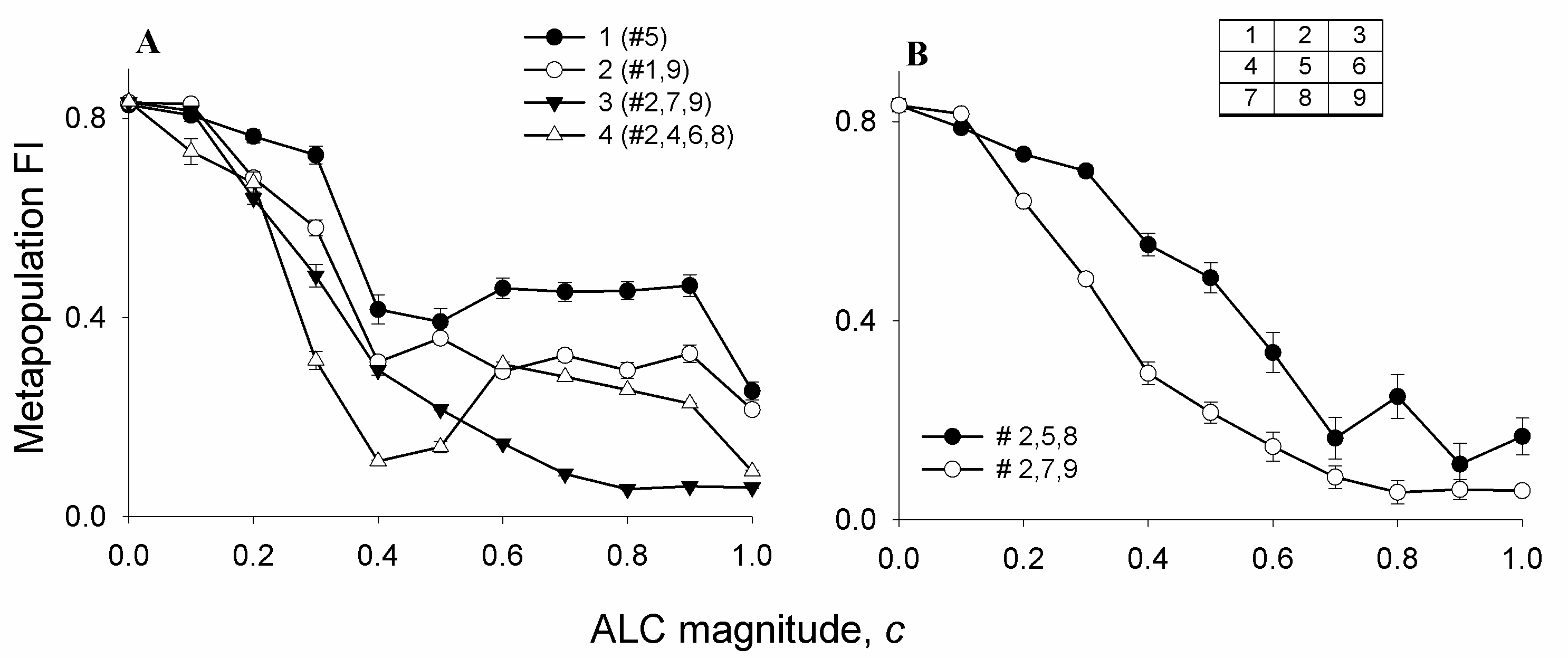

Supplement: Figure S3 — FI of 9-patch 2-D metapopulations for different ALC magnitude ( c ). In these simulations, the 9 subpopulations were arranged on a 3×3 2-D lattice with periodic boundary conditions for migration. The inset gives the identity of the individual subpopulations. Each subpopulation exchanged migrants with four neighbours (above, below, right and left). Thus, subpopulation #5 exchanged migrants with subpopulation # 2,8,6,4 and so on. With periodic boundary conditions, the subpopulations thus inhabit the surface of a 3-D torus. The migration rate, initial population size, r, and K were 0.3, 20, 3.5 and 30 respectively. All other conditions were similar to the 1-D migrations. Each point is a mean of 100 independent simulations and error bars denote the corresponding SEM. a) Metapopulation stability for different number of perturbed subpopulations (identity of subpopulations in bracket) at different values of c. ALC was able to stabilize metapopulations in general, even when applied in only 1/9 subpopulations. However, perturbing too many subpopulations leads to a lesser reduction in global FI. b) Metapopulation stability when three subpopulations are perturbed but in different arrangements. Note that although there is an overall decrease in metapopulation FI, the trends and magnitude of decrease are different. (TIF) [file pone.0105861.s003.tif]

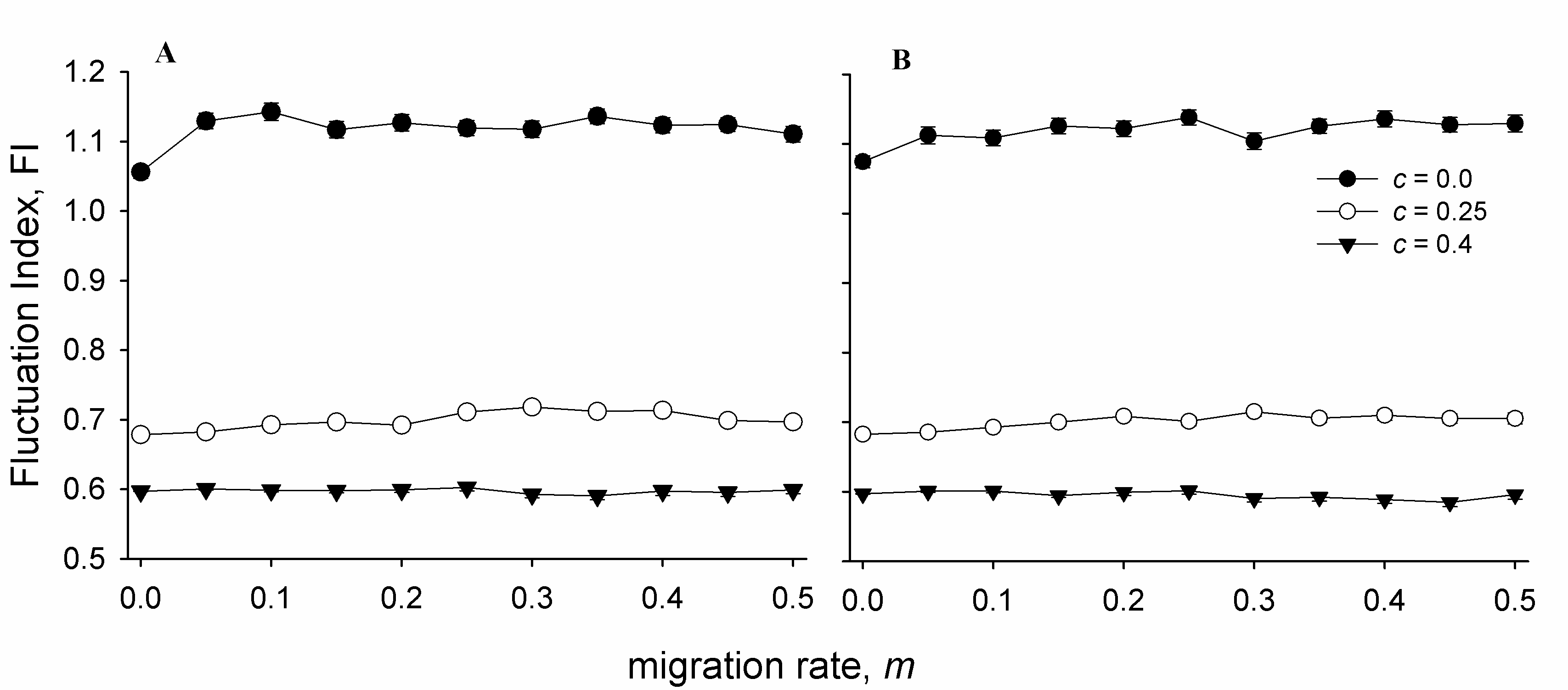

Supplement: Figure S4 — Effect of ALC on two-patch Logistic and Hassell metapopulations. (a) Effects of ALC on metapopulation FI using (a) coupled logistic map (x = r.x(1-x), r = 0.4, x0 = 0.1) and (b) coupled Hassell map (; a = 0.6, b = 10, no = 0.4, r = 40) at different migration rates. Both LALC (c = 0.25) and HALC (c = 0.4) reduces metapopulation FI at all migration rate values tested. Thus qualitatively, the effects of ALC on coupled logistic and Hassell map are comparable to those from Ricker (cf Fig 1A of the main paper). (TIF) [file pone.0105861.s004.tif]

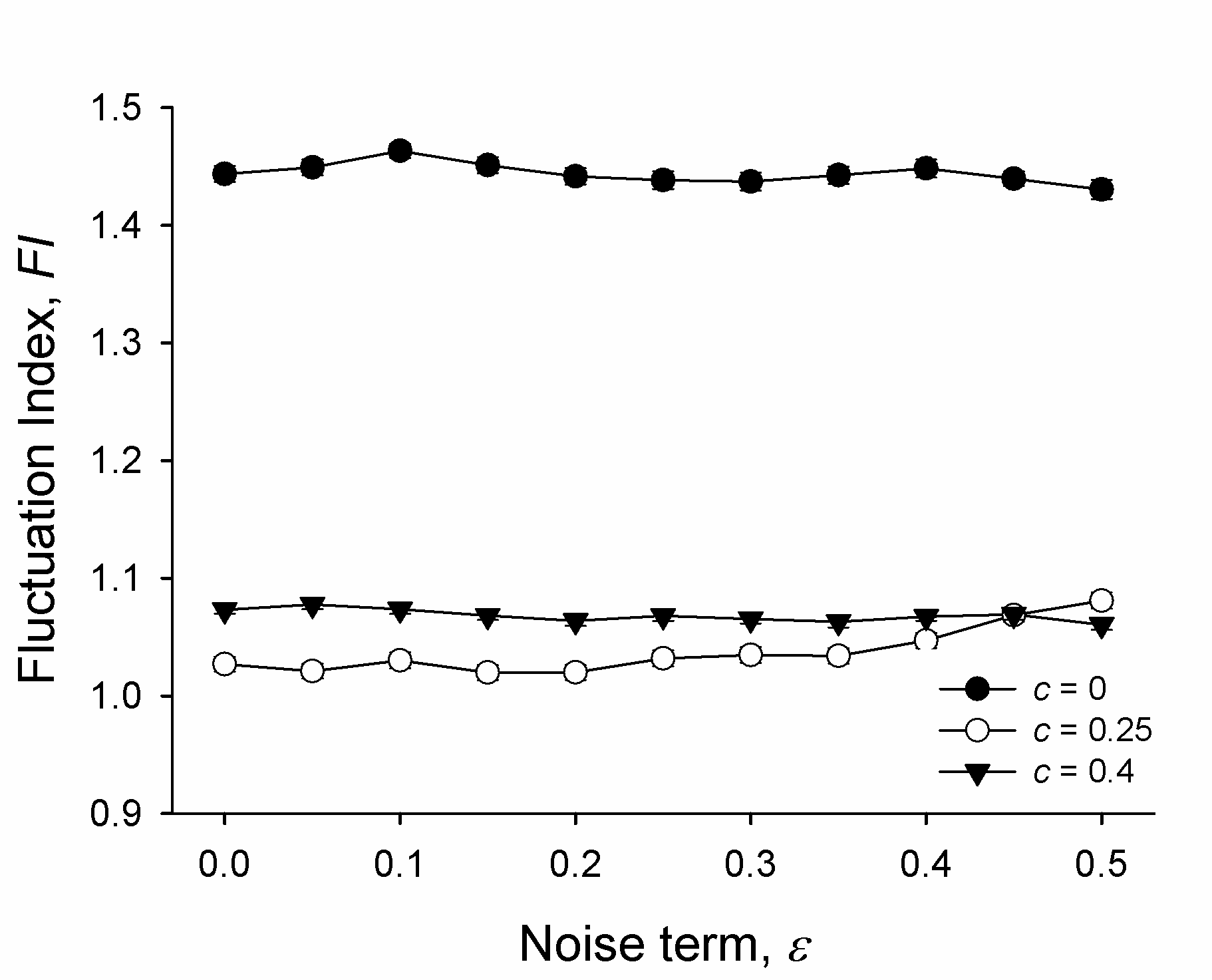

Supplement: Figure S5 — Effects of noise in growth rate on constancy of two-patch metapopulations. In this figure, each metapopulation consists of 2 subpopulations. Noise term (ε) represents the magnitude of noise associated with intrinsic growth rate (r) in the simulations. Both LALC (c = 0.25) and HALC (c = 0.4) are robust to varying degrees of noises. Migration rate, m = 0.3. Each point is a mean of 100 independent simulations. Error bars denote ±SEM and are too small to be visible. (TIF) [file pone.0105861.s005.tif]
